# A novel immune resistance mechanism of melanoma cells controlled by the ADAR1 enzyme

**DOI:** 10.18632/oncotarget.4905

**Published:** 2015-08-21

**Authors:** Gilli Galore-Haskel, Yael Nemlich, Eyal Greenberg, Shira Ashkenazi, Motti Hakim, Orit Itzhaki, Noa Shoshani, Ronnie Shapira-Fromer, Eytan Ben-Ami, Efrat Ofek, Liat Anafi, Michal J. Besser, Jacob Schachter, Gal Markel

**Affiliations:** ^1^ Ella Lemelbaum Institute of Melanoma, Sheba Medical Center, Israel; ^2^ Institute of Pathology, Sheba Medical Center, Israel; ^3^ Talpiot Medical Leadership Program, Sheba Medical Center, Israel; ^4^ Department of Clinical Microbiology and Immunology, Tel Aviv University, Tel Aviv, Israel; ^5^ cCAM Biotherapeutics, Misgav Industrial Park, Misgav, Israel

**Keywords:** melanoma, immune resistance, microRNA, ADAR1, ICAM1

## Abstract

The blossom of immunotherapy in melanoma highlights the need to delineate mechanisms of immune resistance. Recently, we have demonstrated that the RNA editing protein, adenosine deaminase acting on RNA-1 (ADAR1) is down-regulated during metastatic transition of melanoma, which enhances melanoma cell proliferation and tumorigenicity. Here we investigate the role of ADAR1 in melanoma immune resistance.

Importantly, knockdown of ADAR1 in human melanoma cells induces resistance to tumor infiltrating lymphocytes in a cell contact-dependent mechanism. We show that ADAR1, in an editing-independent manner, regulates the biogenesis of miR-222 at the transcription level and thereby Intercellular Adhesion Molecule 1 (ICAM1) expression, which consequently affects melanoma immune resistance. ADAR1 thus has a novel, pivotal, role in cancer immune resistance. Corroborating with these results, the expression of miR-222 in melanoma tissue specimens was significantly higher in patients who had no clinical benefit from treatment with ipilimumab as compared to patients that responded clinically, suggesting that miR-222 could function as a biomarker for the prediction of response to ipilimumab.

These results provide not only novel insights on melanoma immune resistance, but also pave the way to the development of innovative personalized tools to enable optimal drug selection and treatment.

## INTRODUCTION

Malignant melanoma, arising from pigment producing melanocytes, is among the most aggressive and treatment-resistant human cancers. The incidence of melanoma in Caucasian populations has been increasing at a higher rate than any other malignancy [1].

Metastatic melanoma responds poorly to conventional chemotherapies and predicts poor survival rates. Melanoma is considered as an immunogenic tumor, expressing a variety of tumor associated antigens [2, 3]. In 2011 the FDA approved anti- cytotoxic T lymphocyte-associated protein 4 (CTLA4) mAb (ipilimumab) for the indication of metastatic melanoma, based on significant improved overall survival in Phase III trials [4, 5]. Recent clinical trials with PD1 [6–8] or PD-L1 blocking antibodies [9] showed impressive effects, leading to FDA approval of anti-PD1 drugs pembrolizumab and nivolumab in 2014. Combination of ipilimumab and PD1 blockade yield dramatic effects with ∼80% 2-year survival, but with very high toxicity [10]. In addition, adoptive cell transfer using autologous tumor infiltrating lymphocytes (TILs) has shown impressive results in about 40% of the patients [11–13]. Since metastatic melanoma uses many mechanisms to escape the immune system [2], delineation of mechanisms involved in melanoma immune-resistance is of cardinal importance.

RNA editing is a post-transcriptional mechanism through which RNA sequences are directly altered. Specific adenosine-to-inosine (A-to-I) editing is catalyzed by members of the family of adenosine deaminases that act on RNA (ADARs). ADARs convert adenosines to inosines in double-stranded RNA (dsRNA) substrates by hydrolytic deamination of the adenine base. In mammals, three ADAR proteins have been identified: ADAR1 and ADAR2 are detected in many tissues, whereas ADAR3 is brain-specific. ADAR1 has two isoforms as a result of alternative splicing: the longer form (p150) is modulated mostly by interferon-alpha (IFNα) and found both in nucleus and cytoplasm, while the shorter form (p110) is constitutively expressed, but only present in the nucleus [14]. The splicing and translational machineries recognize inosine (I) as guanosine (G), resulting in significant biological effects. Rare events of editing in coding regions may result in amino acid substitutions [15], while editing in non-coding regions might affect splicing, RNA stabilization and nuclear retention [16, 17]. Furthermore, editing of non-coding RNAs affects their biogenesis or alters their target gene specificity [18–20].

We have recently reported on a significant decrease in ADAR1 expression in ∼65% of metastatic melanoma specimens compared to melanocytes [21]. This down regulation enhances the proliferation of melanoma cells, probably by controlling the biogenesis pathway of miRNAs, and thereby their entire expression profile [21]. Little is known about the role of RNA editing and ADARs in immune function. Recent studies have identified ADAR1 as an essential regulator of hematopoietic stem cell maintenance and suppressor of interferon signaling [22] and as a dominant player in the regulation of primary T lymphocytes function during acute transplant rejection [23].

In the present study we investigate the role of ADAR1 in the regulation of melanoma immune resistance. We show that ADAR1, in an editing-independent manner, transcriptionally regulates the biogenesis of miR-222 and thereby Intercellular Adhesion Molecule 1 (ICAM1) expression, which consequently affects melanoma immune resistance. Moreover, we show that miR-222 expression in melanoma may serve as a biomarker for prediction to response to immunotherapy, such as ipilimumab.

## RESULTS

### Regulation of melanoma immune resistance to T cells by ADAR1

**Figure 1:**
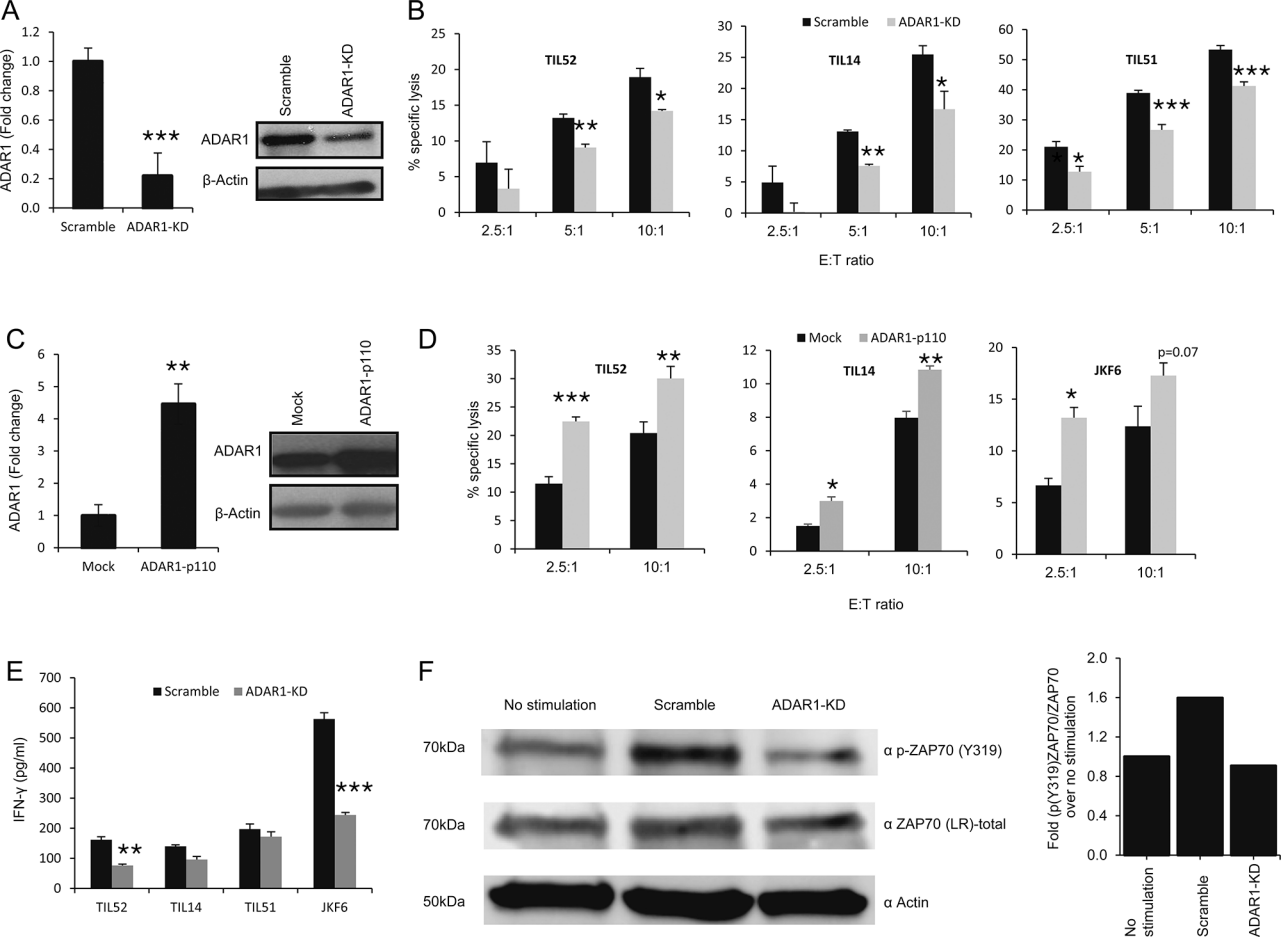
ADAR1 regulates melanoma immune resistance **A, C.** ADAR1 mRNA levels were assessed by qRT-PCR and normalized to GAPDH expression. ADAR1 protein expression was determined by WB using ADAR1 polyclonal antibody. **B, D.** 624mel ADAR1-KD and Scramble cells (B) 624mel ADAR1-p110 and Mock cells (D) were co-incubated in different E:T ratios with various TIL cultures (i.e., TIL52, TIL51 or TIL14) or TIL clone (JKF6) overnight or 9 h, respectively. Specific lysis of melanoma cells was assessed by LDH release. **E.** 624mel ADAR1-KD and Scramble cells were co-incubated with various TIL cultures or clones for 5 h. IFN-γ secretion was evaluated by ELISA. **F.** 624mel ADAR1-KD or Scramble cells were mixed with TIL14; stimulations were carried for 10min at 37°C. Then, cells were lysed followed by WB, to determine expression of p-ZAP70(Y319), total ZAP70 and actin. Quantification of data was performed as folds of pZAP70(Y319)/ZAP70 over no stimulation. Experiments were performed three times in triplicates. WB figures show one representative experiment.

ADAR1 expression was stably knocked-down in 624mel melanoma cells (ADAR1-KD) or with scrambled sequence as control (Scramble) (Figure 1A). In addition, 624mel melanoma cells were stably transfected with ADAR1-p110 or empty vector as control (Mock) (Figure 1C). ADAR1-manipulated cells were used as target cells for primary TIL cultures (TIL14, TIL51 and TIL52) and clones (JKF6). ADAR1-KD cells were significantly more resistant to killing by all TIL cells, in all E:T ratios tested, as compared to the control (Figure 1B). Importantly, over-expression of ADAR1 rendered the melanoma cells more sensitive to all TIL cells tested in different E:T ratios (Figure 1D). Similar results were obtained with additional melanoma and TIL cells (Supplementary Figure S1A–S1D).

Previous studies showed that TIL anti-melanoma activity is HLA-A2 restricted [24]. Melanoma cells, TIL clone and polyclonal populations used in this study share the HLA-A2 allele, as described in Materials and Methods section. Blocking of HLA-A2 with blocking mAb abrogated completely the killing of HLA-A2(positive) 526mel and 624mel cells by JKF6, TIL14 and TIL52, but not of HLA-A2(negative) 938mel cells (Supplementary Figure S2A), attesting that the activity of TILs used in this study is HLA-A2 specific.

To rule out the possibility that ADAR1 enhances endogenous melanoma cell resistance to cytotoxicity, its effect on spontaneous apoptosis was tested by staining ADAR1-p110 and control cells with AnnexinV and PI. No effect of ADAR1 on spontaneous apoptosis could be observed (Supplementary Figure S3A). Further, we have previously demonstrated that knockdown of ADAR1 has no effect on induced apoptosis [21]. In addition, IFN-γ release was measured concomitantly to cytotoxicity. A substantially reduced secretion of IFN-γ by at least some of the TILs was demonstrated when co-incubated with ADAR1-KD cells as compared to control (Figure 1E). Finally, we tested the phosphorylation of ZAP-70 (ζ-associated protein of 70 kDa) within TIL cells as a direct measurement for T cell activation [25]. Indeed, enhanced phosphorylation of ZAP-70 was observed when TIL14 cells were incubated with control melanoma cells, as compared to basal levels (Figure 1F). Remarkably, phosphorylated ZAP-70 level was significantly lower when TIL14 cells were incubated with ADAR1-KD cells as compared to control cells (Figure 1F).

Altogether, these combined observations strongly suggest that ADAR1 in the melanoma cells protects melanoma cells by affecting T-cell activation, and not by altering inherent cell resistance.

### Regulation of immune resistance by ADAR1 depends on cell-cell interactions

**Figure 2:**
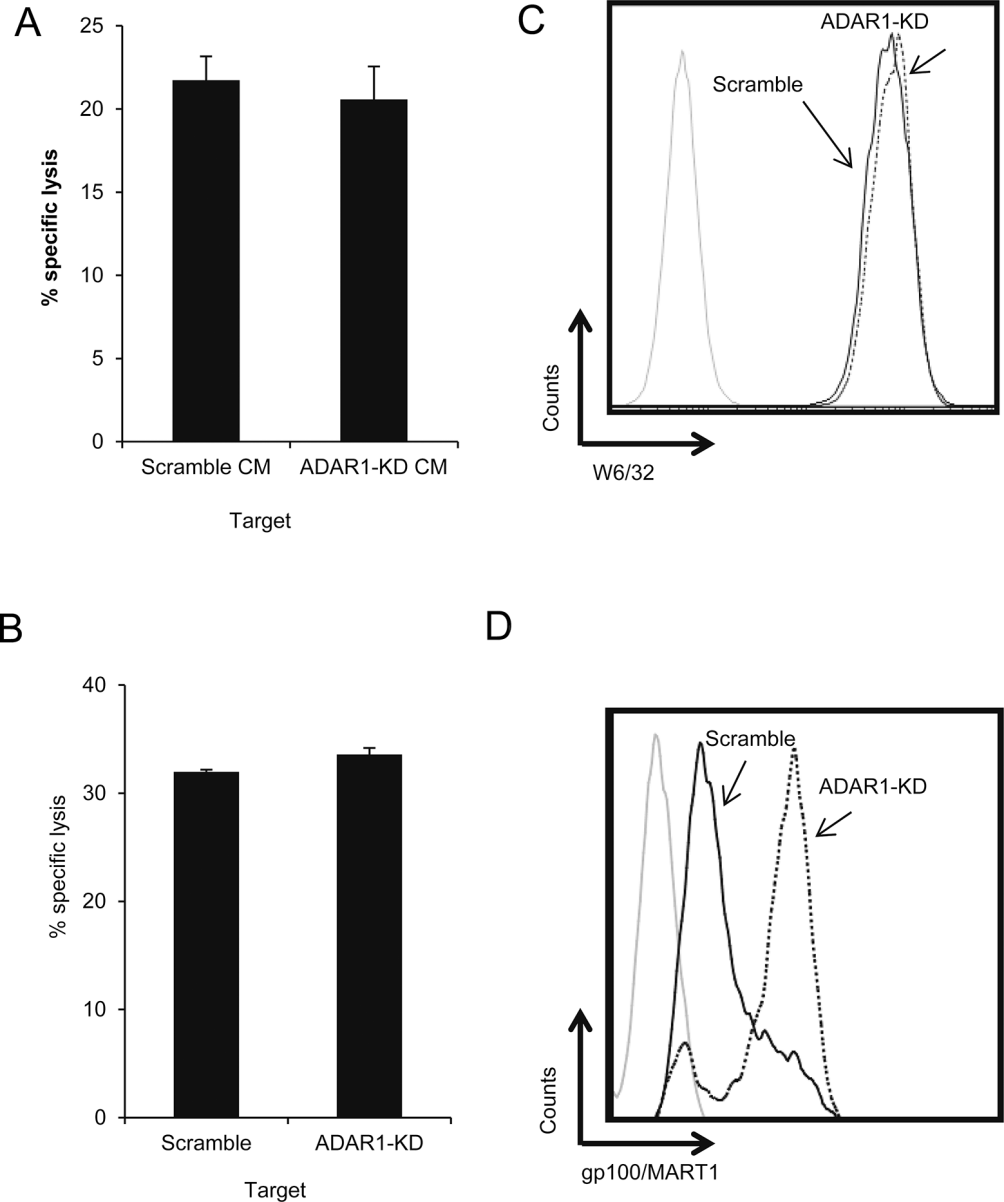
ADAR1-mediated regulation of melanoma immune resistance is dependent on cell-cell interaction **A.** TIL52 were pre-incubated with conditioned media (CM) from 624mel ADAR1-KD or Scramble cells. After 1 h, the cells were co-incubated overnight with non-manipulated 624mel cells (E:T – 15:1). Specific lysis of 624mel cells was assessed by flow cytometry. **B.** 624mel ADAR1-KD or Scramble cells were loaded in the upper well of a transwell chamber. Non-manipulated 624mel cells were co-incubated overnight with TIL14 (E:T – 15:1) in the transwell lower well. Specific lysis of 624mel cells was assessed by flow cytometry. **C.** ADAR1-KD (dotted line) and Scramble (black line) cells were stained with anti-HLA Class I antibody (W6/32). Expression was analyzed by flow cytometry. **D.** ADAR1-KD (dotted line) and Scramble (black line) cells were stained with anti-melanoma antibody gp100/MART1). Expression was analyzed by flow cytometry. Experiments were performed three times in triplicates. Flow cytometry figures show one representative experiment.

Conditioned media (CM) were obtained from 300,000 624mel ADAR1-KD or Scramble cells pre-cultured for 24 h. TILs were pre-incubated for 1 h with the CM. Then, non-manipulated melanoma cells were added and co-incubated overnight. There were no significant differences in killing activity of TIL52 among the various treatments (Figure 2A). Similar results were obtained with other cells and following 5 h incubation (Supplementary Figure S3B, S3C). To overcome concerns regarding stability and decay of soluble factors, we performed a cytotoxicity assay using a two chamber transwell system, allowing passage of solutes, but not cell migration. ADAR1-KD or Scramble cells were seeded in the upper well, while TILs and non-manipulated CFSE-labeled melanoma cells were co-incubated in the lower well. There were no significant differences in the killing activity of TIL14 among all tested setups (Figure 2B). Similar results were obtained with other cells and following 5 h incubation (data not shown). FACS analysis confirmed that the melanoma cells seeded in the upper well did not migrate into the lower well (data not shown). These results point on cell-contact dependent mechanism.

MHC class I expression among ADAR1-KD and Scramble cells was similar (Figure 2C). The expression of melanosomal proteins (gp100/MART1) was actually higher in ADAR1-KD cells as compared to Scramble cells in the 624mel cell system (Figure 2D), but in other melanoma cell systems there were no significant differences in the expression of these proteins (Supplementary Figure S1E, S1F). These observations suggest that altered antigenic recognition is a less probable explanation.

### ADAR1 regulates ICAM1 protein expression

**Figure 3:**
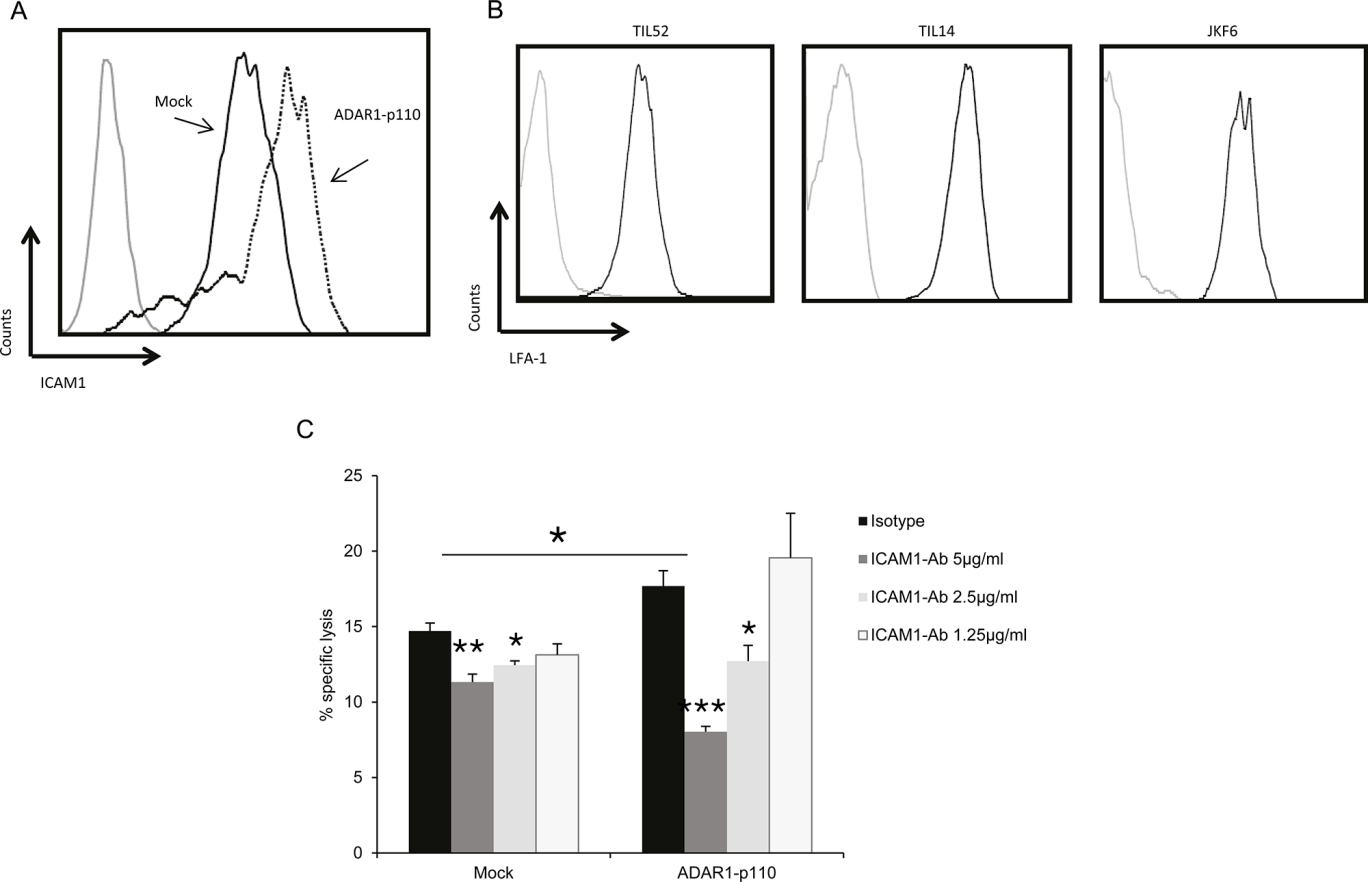
ADAR1 regulates ICAM1 expression, which contributes to TIL mediated killing **A.** ICAM1 protein expression in ADAR1-p110 (dotted line) and Mock cells (black line) was analyzed by extracellular flow cytometry staining using ICAM1 monoclonal antibody. **B.** LFA-1 protein expression in TIL52, TIL14 and JKF6 was analyzed by extracellular flow cytometry staining using human LFA-1 alpha antibody. **C.** ADAR1-p110 and Mock cells were incubated with different concentrations of IgG1 control (isotype) or ICAM1 antibody. After 1 h, cells were co-incubated with JKF6 for 9 h (E:T - 15:1). Specific lysis of melanoma cells was assessed by LDH release. Experiments were performed three times in triplicates. Flow cytometry figures show one representative experiment.

The effect of ADAR1 on immune resistance was evident in several T cell cultures with different antigenic specificities (Figure 1 and Supplementary Figure S1). This, combined with the evidence pointing to cell-contact dependent mechanism, implies on an adhesive element, such as ICAM1. Binding of ICAM1 to the CTL integrin lymphocyte function-associated antigen-1 (LFA-1) is an essential step in the formation of the immune synapse [26] and facilitates T cell activation [27]. Indeed, over-expression of ADAR1 increased ICAM1 expression as compared to Mock-transfected cells (2.2-fold increase) (Figure 3A). Expression of LFA-1 on TIL52 and TIL14 bulk cultures and JKF6 clone was confirmed (Figure 3B). Remarkably, the enhanced killing of ADAR1-transfected melanoma cells was significantly reduced by a blocking anti-ICAM1 mAb, in a dose dependent manner (Figure 3C), while only mild reduction in killing of control cells was observed following ICAM1 blocking. This indicates on a major role of ICAM1 as an effector molecule of ADAR1 in mediating this phenomenon.

### ADAR1 regulates miR-222 expression

**Figure 4:**
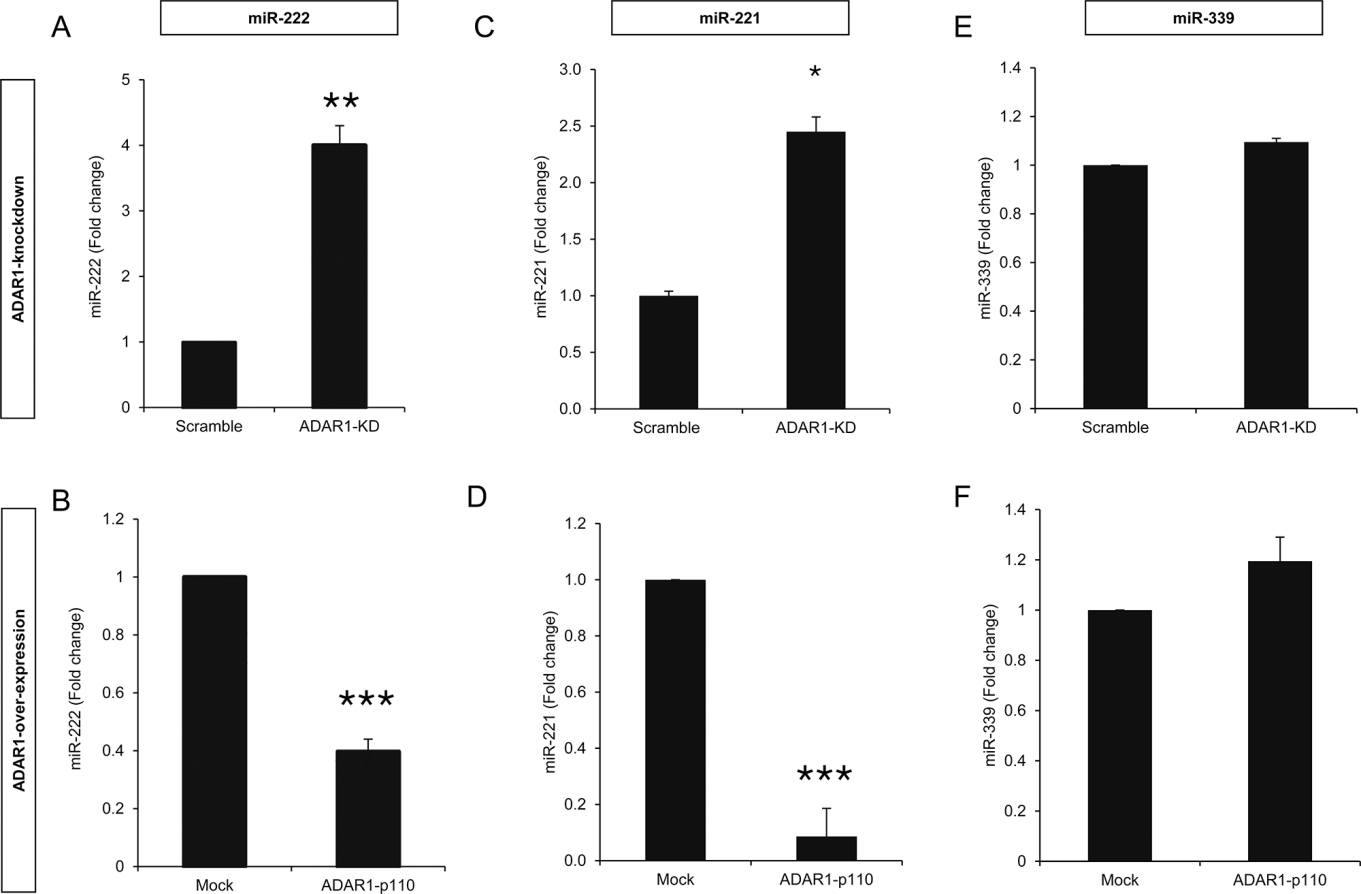
ADAR1 regulates miR-222 and miR-221 expression Expression levels of hsa-miR-222, hsa-miR-221 and hsa-miR-339 in ADAR1-KD and Scramble cells **A, C, E.** and ADAR1-p110 and Mock cells **B, D, F.** were assessed by qRT-PCR and normalized to U6 expression. All experiments were performed three times in triplicates.

**Figure 5:**
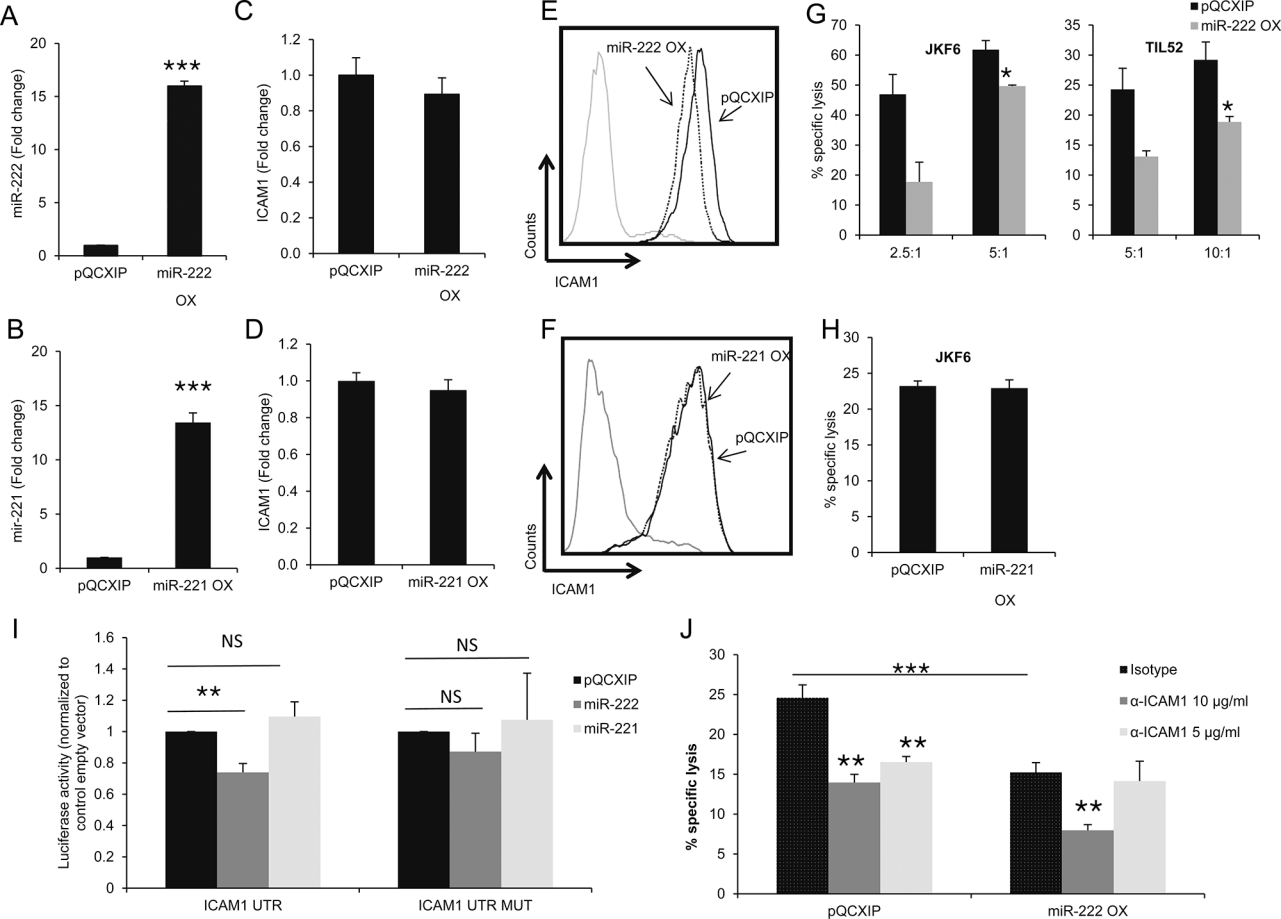
miR-222 suppresses ICAM1 expression at the protein level and enhances immune resistance **A–B.** 624mel cells were transfected with miR-222 precursor, mir-221 precursor or control (pQCXIP) plasmid. Expression levels were assessed by qRT-PCR and normalized to U6 expression. **C–D.** ICAM1 mRNA levels in miR-222 OX, miR-221 OX and pQCXIP cells were assessed by qRT-PCR and normalized to GAPDH expression. **E–F.** ICAM1 protein expression in miR-222 OX (dotted line) (E), miR-221 OX (dotted line) (F) and pQCXIP (black line) cells was analyzed by extracellular flow cytometry staining. **G–H.** miR-222 OX or miR-221 OX cells and pQCXIP cells were co-incubated in different E:T ratios with JKF6 or TIL52 for 5 h or overnight. Specific lysis of melanoma cells was assessed by flow cytometry or LDH release. **I.** 293T cells were co-transfected with miR-222, mir-221 or control (pQCXIP empty vector) constructs and with ICAM1 UTR or ICAM1 UTR MUT which is mutated at the predicted binding site of miR-221 and miR-222. Dual luciferase assay was carried out and Renilla luciferase activity was measured and normalized to the firefly constitutive luciferase activity. Relative Luciferase activity was normalized to the Luciferase activity of control vector. NS denotes “not significant”. Experiment was performed three times in sixplicates. **J.** miR-222 OX and pQCXIP cells were incubated with different concentrations of IgG1 control (isotype) or ICAM1 antibody. After 1 h, cells were co-incubated with JKF6 for 9 h (E:T - 15:1). Specific lysis of melanoma cells was assessed by LDH release. Experiments were performed three times in triplicates. Flow cytometry figures show one representative experiment.

It was previously suggested that ICAM1 is regulated by miR-222 and miR-339 in colorectal cancer cells and glioma cells [28] and by miR-221 in cholangiocytes [29]. Remarkably, knockdown of ADAR1 resulted in a 4-fold up-regulation of hsa-miR-222 (Figure 4A), while over-expression of ADAR1 reduced hsa-miR-222 expression by more than 2-fold (Figure 4B). Similar results were obtained for hsa-miR-221 (Figures 4C, 4D) while the expression level of hsa-miR-339 did not change (Figures 4E, 4F). 624mel cells were stably transfected with miR-222 precursor (miR-222 OX; Figure 5A), miR-221 precursor (miR-221 OX; Figure 5B) or empty vector (pQCXIP). All cells similarly expressed ICAM1 mRNA (Figures 5C, 5D). At the protein level, over-expression of miR-222 led to the down-regulation of ICAM1 (Figure 5E), but not of another adhesion molecule, CEACAM1 (Supplementary Figure S2B). Surprisingly, over-expression of miR-221 had no effect on ICAM1 (Figure 5F). Functionally, over-expression of miR-222, but not miR-221, rendered the melanoma cells more resistant to TIL mediated killing, as compared to control (Figures 5G, 5H). To confirm direct regulation of ICAM1 by miR-222 but not miR-221, we performed a set of dual luciferase assays. 293T cells were co-transfected with miR-222, miR-221 or pQCXIP empty vector as control and with ICAM1 UTR, ICAM1 UTR MUT or psiCheck2 empty vector. Forced expression of miR-222 with ICAM1 UTR construct significantly inhibited the luciferase activity, while the inhibitory effect was abolished when the ICAM1 UTR MUT construct was tested (Figure 5I). miR-221 did not affect the luciferase activity and was similar to that observed with the control pQCXIP empty vector (Figure 5I). These results reinforce our previous results (Figure 5E, 5F), suggesting that ICAM1 is a direct target of miR-222 but not of miR-221.

To further test the role of ICAM1 in the immune resistance conferred by miR-222, we performed cytotoxicity assays using blocking α-ICAM1 mAb. While 5 μg/ml of α-ICAM1 significantly reduced the killing of control cells as compared to isotype control, the antibody had no significant effect on the killing rate of miR-222-OX cells. Interestingly, at this antibody concentration, blocking of ICAM1 yielded a similar inhibitory effect as miR-222 over-expression (Figure 5J). Only when we used an even higher concentration of 10 μg/ml α-ICAM1, a reduction in killing rates was observed also in miR-222-OX cells (Figure 5J).

These results suggest that ADAR1 controls ICAM1 expression at the translation level via miR-222, and thereby the immune resistance phenotype of melanoma cells. Moreover, it is suggested that despite identical seed region, miR-222 and miR-221 have distinct target gene profiles.

### ADAR1 regulates immune resistance independently of RNA-editing

**Figure 6:**
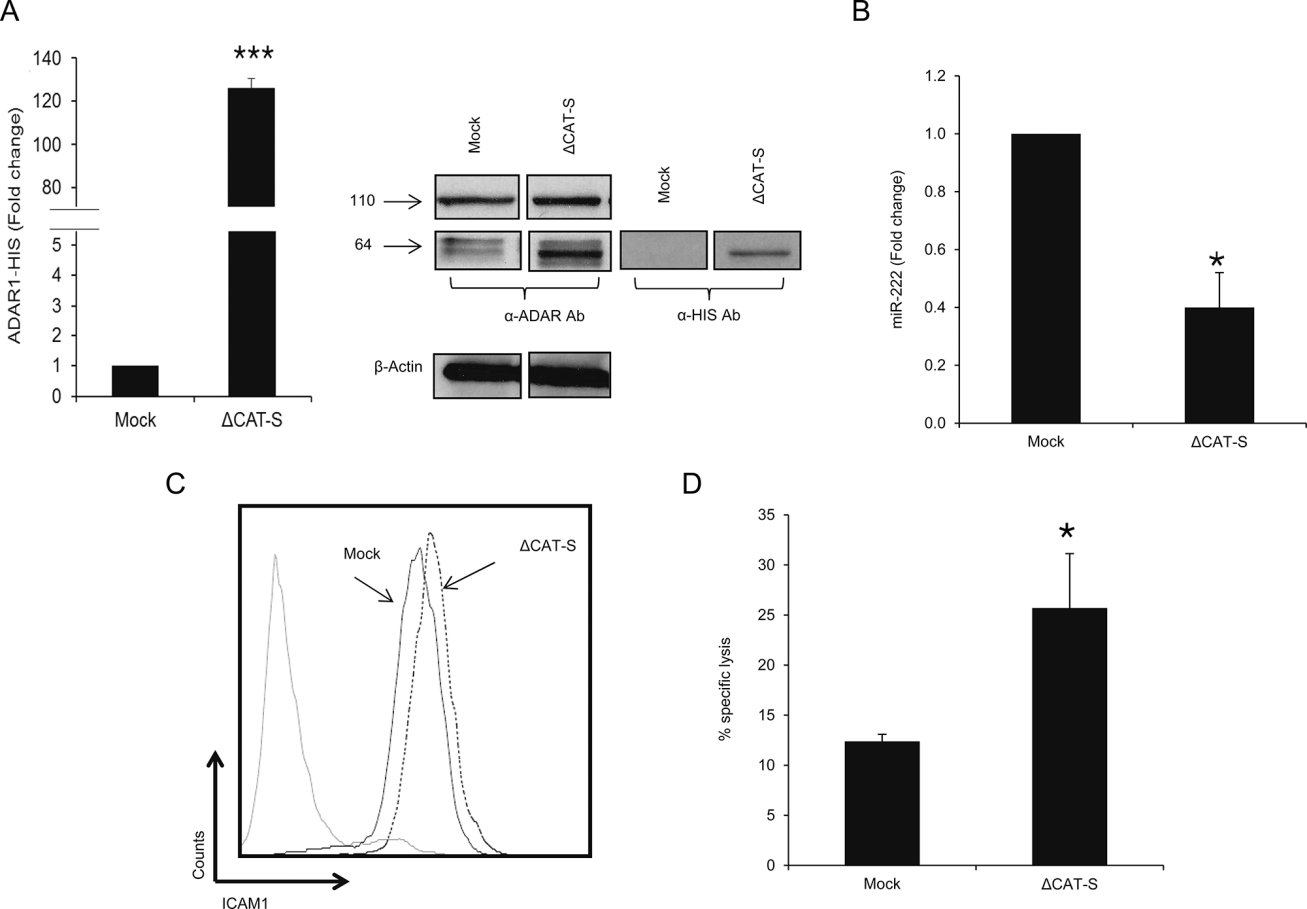
ADAR1 mediated-regulation is editing independent **A.** ADAR1 His-tagged mRNA levels were assessed by qRT-PCR and normalized to GAPDH expression. Protein expression of ADAR1 His-tagged constructs was determined by WB using ADAR1 polyclonal antibody and polyHistidine monoclonal antibody (cut from different regions in the same membrane). **B.** Expression levels of hsa-miR-222 in ΔCAT-S and Mock cells were assessed by qRT-PCR and normalized to U6 expression. **C.** ICAM1 protein expression in ΔCAT-S (dotted line) and Mock cells (black line) was analyzed by extracellular flow cytometry staining. **D.** ΔCAT-S and Mock cells were co-incubated with JKF6 for 5 h (E:T - 15:1). Specific lysis of melanoma cells was assessed by flow cytometry. Experiments were performed three times in triplicates. WB and flow cytometry figures show one representative experiment.

The role of ADAR1 as an RNA editing enzyme is well documented [14, 30], however there is very little evidence on its functions independently of its enzymatic activity [21, 31, 32]. Sequencing of PCR-amplified pri-miR-222, from which the mature miR-222 is derived, did not reveal any A-to-I RNA editing sites or any sequence differences among the various ADAR1-manipulated cells. To test the involvement of A-to-I RNA editing in this phenomenon, a His-tagged ADAR1 construct of ∼64 kDa lacking the deaminase domain (ΔCAT-S) was generated and stably transfected into 624mel cells (Figure 6A). Expression levels of hsa-miR-222 were significantly lower (Figure 6B) and ICAM1 protein expression was higher (Figure 6C), as compared to control. Moreover, cytotoxicity assays confirmed that transfection with ΔCAT-S, rendered the cells significantly more sensitive to TIL-mediated killing (Figure 6D). Importantly, the effects exerted by ΔCAT-S were similar to those observed with the full ADAR1-p110 protein, suggesting that ADAR1 regulates immune resistance independently of RNA editing.

### ADAR1 transcriptionally regulates miR-222

**Figure 7:**
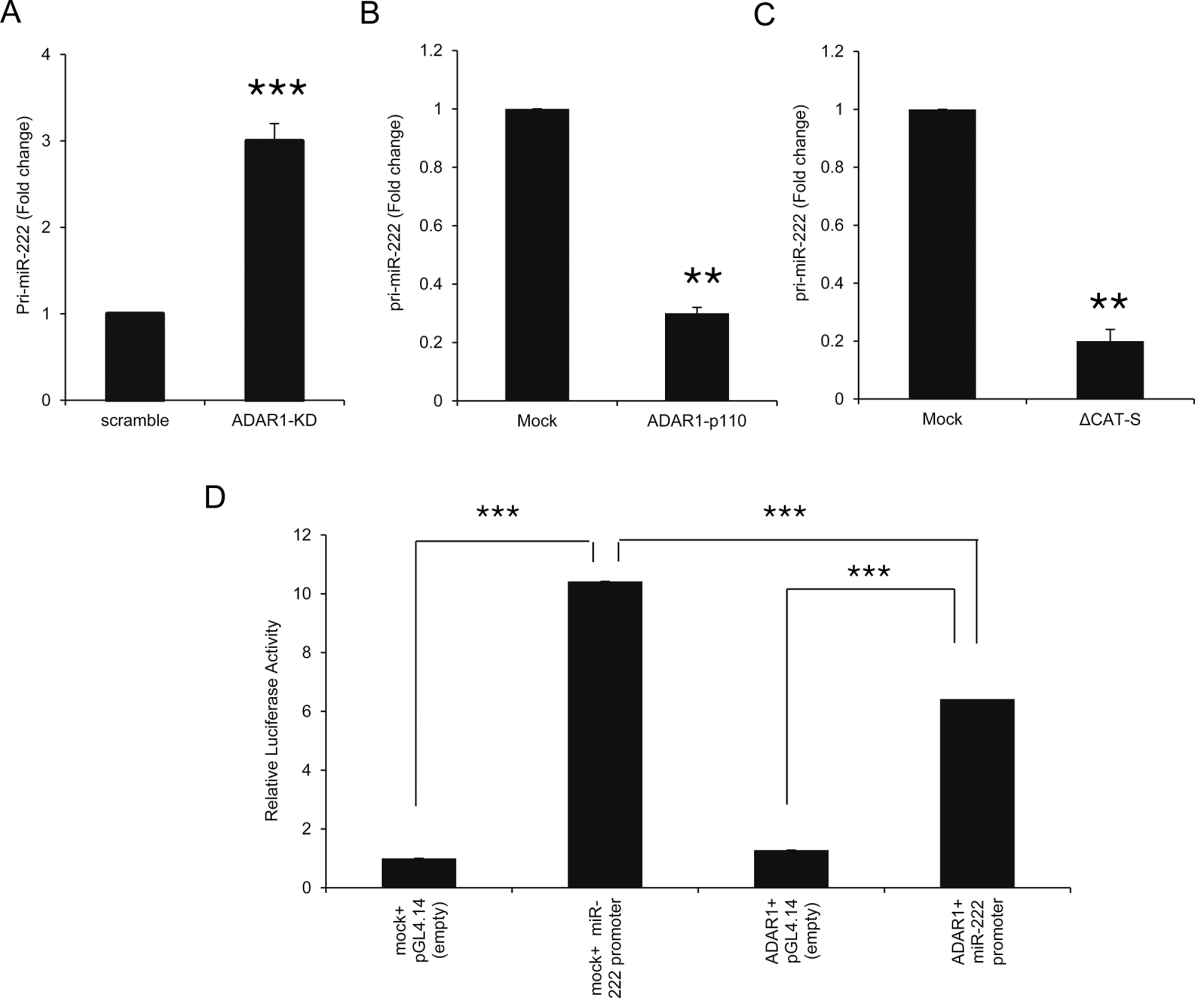
ADAR1 transcriptionally regulates miR-222 processing at the pri-miR level **A–C.** Expression levels of pri-miR-222 in ADAR1-KD and scramble cells (A); ADAR1-p110 and Mock cells (B); and ΔCAT-S and Mock cells (C) were assessed by qRT-PCR and normalized to HPRT expression. Experiments were performed three times in triplicates. **D.** miR-222 putative promoter was cloned into pGL4.14 vector and luciferase assays were performed with ADAR1-p110 or Mock constructs. Relative luciferase activity was calculated relative to control (i.e., Mock in the presence of pGL4.14 empty vector). Experiment was performed in sixplicates. Representative experiment out of three.

The biogenesis of miRNAs is a multistep process tightly controlled by several enzymes and complexes [33]. We have recently shown that ADAR1 regulates the miRNA biogenesis pathway [21]. Expression levels of pri-miR-222 were significantly higher in ADAR1-KD cells (Figure 7A) and lower in ADAR1-p110 (Figure 7B), as compared to controls. These observations were similar to the results of the mature miR-222 expression (Figures 4A, 4B). Moreover, the expression of pri-miR-222 was lower in ΔCAT-S cells as compared to control (Figure 7C), suggesting an RNA-editing independent regulation.

Luciferase assays demonstrated reduced activity of miR-222 putative promoter when co-transfected with ADAR1 as compared to Mock construct (Figure 7D), suggesting that ADAR1 affects the transcription of miR-222 precursors. Direct binding of ADAR1 to pri-miR-222 could not be demonstrated over wide range of experimental parameters of PCR amplification after immunoprecipitation of ADAR1 (data not shown).

### miR-222 expression predicts response to ipilimumab

Ipilimumab is an immune checkpoint inhibitor that potentiates immune responses [34] and is prescribed for metastatic melanoma patients. The drug was approved based on clinical benefit to a subpopulation of the patients, but there is still no biomarker that can predict who will benefit from the treatment. We performed a miRNA expression profile analysis of melanoma tissue specimens derived from patients with metastatic melanoma that showed clinical benefit (CB, *n* = 5) after ipilimumab treatment versus those who did not (NB, *n* = 8). Samples were taken pre-treatment and RNA was purified from FFPE slides. miR-222 was the only miR, out of the 1105 tested, that was differentially expressed (fold change > = 2) in a statistically significant manner. The expression of hsa-miR-222 in melanoma tissues of NB patients was 2.3-fold higher (*p*-value = 0.001) than in CB patients (Table 1). Similar results were obtained when validating the expression of miR-222 by qRT-PCR in 22 melanoma tissues (CB, *n* = 7 and NB, *n* = 15), suggesting that miR-222 expression may be useful as a marker for prediction to response to ipilimumab.

**Table 1:** miR expression in melanoma tumors derived from ipilimumab-treated patients^1^

microRNA	*p*-value	Fold change (NB vs CB)
**hsa-miR-222**	**0.001**	**2.24**
hsa-miR-1292	0.020	1.27
hsa-miR-23a-star	0.022	1.27
hsa-miR-140-5p	0.009	1.21
hsa-miR-1273d	0.022	1.16
hsa-miR-539	0.044	1.14
hsa-miR-145-star	0.049	1.14
hsa-miR-30c-1-star	0.039	1.14
hsa-miR-3194	0.039	1.13
hsa-miR-545-star	0.004	1.13
hsa-miR-593-star	0.041	1.12
hsa-miR-2114	0.010	1.12
hsa-miR-27b-star	0.038	1.11
hsa-miR-215	0.020	1.10
hsa-miR-129-3p	0.029	1.10
hsa-miR-549	0.011	1.09
hsa-miR-23b-star	0.004	1.08
hsa-miR-92a-2-star	0.044	1.07
hsa-miR-2117	0.042	–1.08
hsa-miR-146b-3p	0.017	–1.08
hsa-miR-4330	0.036	–1.09
hsa-miR-454	0.020	–1.09
hsa-miR-338-5p	0.049	–1.10
hsa-miR-1303	0.044	–1.10
hsa-miR-548a-5p	0.036	–1.10
hsa-miR-326	0.025	–1.11
hsa-miR-369-3p	0.020	–1.12
hsa-miR-4325	0.007	–1.12
hsa-miR-519e	0.023	–1.13
hsa-miR-1244	0.041	–1.13
hsa-miR-586	0.024	–1.14
hsa-miR-562	0.016	–1.15
hsa-miR-590-5p	0.016	–1.17

1 Only statistically significant (*t* test, *P* value < 0.05) differences are shown.

We next evaluated the rate of TILs and ICAM1 expression in these 22 melanoma specimens. We could not observe any significant differences between the groups in lymphocytes infiltration (positive infiltration in 86% and 93% of CB and NB patients, respectively) and spatial scattering (brisk in 57% and 67% of CB and NB patients, respectively). The median of ICAM1 intensity staining was 2 and 1 for CB and NB, respectively. Percent of samples with high ICAM1 expression (scored 2+3) was 71% and 40% for CB and NB, respectively. Finally, percent of samples with > 50% of tumor cells expressing ICAM1 was 43% and 20% for CB and NB, respectively. However, while ICAM1 staining results seem to support the mechanistic data, none of them reached statistical significance, probably due to the small sample size.

## DISCUSSION

It is well established that melanoma is considered as one of the most immunogenic tumors, expressing a variety of tumor associated antigens. It has been suggested that the immune response plays an important role in the natural history of the disease, as evidenced by infiltration of lymphocytes into the tumor and spontaneous regression of primary melanomas [2, 35]. Yet, metastatic melanoma employs several, not fully understood, mechanisms to escape immune surveillance.

We have recently shown that ADAR1 is commonly down-regulated in metastatic melanoma [21]. Here we show that down-regulation of ADAR1 renders melanoma cells more resistant to TIL-mediated killing, in all E:T ratios tested, which may partially explain why metastatic melanoma tends to evade the immune system. Tumor cells can escape immune surveillance by various mechanisms: 1) tumor-secreted soluble factors; 2) impaired expression of MHC-I or melanoma antigens; 3) deregulation of adhesion and co-stimulating molecules; 4) resistance to apoptosis; and 5) recruitment of immune suppressive cells to the tumor microenvironment [36–38]. We exclude soluble factors and altered expression of MHC-I molecules or melanoma antigens (Figures 2, Supplementary S1E, S1F) as mechanisms for immune resistance following ADAR1 down-regulation. It should be noted that in the 624mel cell system only, ADAR1-KD enhanced the expression levels of gp100/MART1, but still these cells were more resistant to TIL-mediated killing (Figure 2). ADAR1 has no effect on spontaneous or induced apoptosis (Supplementary Figure S3A, [21]). The results hint that resistance depends on cell-cell interaction, pointing to the down-regulation of co-stimulatory or adhesion molecules. Indeed, ICAM1 expression, an adhesion molecule, is controlled by ADAR1. ICAM1-LFA1 interactions are essential for formation of tumor-T-cell immunological synapse [26]. Blocking of ICAM1 in ADAR1-overexpressing cells diminished the enhanced sensitivity to killing, in a dose-dependent manner, supporting the idea that ADAR1-mediated immune resistance is attributed to loss or down-regulation of adhesion molecules, such as ICAM1. Hamai et al further emphasized the role of ICAM1 in immune resistance by showing that reduced expression of ICAM1 in metastatic melanoma, as compared to primary melanoma, was associated with decreased PTEN activity and activation of PI3K/AKT pathway, leading to reduced apoptosis [39]. The reduced IFN-γ release by TIL and reduced phosphorylation of ZAP-70 following incubation with ADAR1-KD confirm that knockdown of ADAR1 in target cells protects them from T cells by reducing T cell activation and not due to altered inherent target cell resistance. The effect of ADAR1-downregulation on tumor microenvironment should be investigated in future studies.

ADARs convert adenosines to inosines in dsRNA substrates [14], including double-stranded miRNAs precursors [18, 40, 41]. In light of the large number of proteins targeted by miRNAs, including cell adhesion molecules [42], we focused on miRNAs that potentially target ICAM1. Recent reports indicate that miR-221, miR-222 and miR-339 directly target ICAM1 [28, 29, 43]. We show that ADAR1 affects miR-221 and miR-222 expression but not miR-339 expression. The miR-221/222 cluster is over-expressed in many types of cancer including pancreatic cancer [44], papillary thyroid carcinoma [45], prostate carcinoma [46] and metastatic melanoma [47]. In line with previous reports [28, 43], transfection of miR-222 precursor into melanoma cells reduced ICAM1 at the post-transcriptional level (Figure 5E) and enhanced the resistance to TIL-mediated killing (Figure 5G), similar to the effect of ADAR1 knockdown. These findings concur with a previous work showing that inhibition of miR-222 in glioma cells leads to recovery of ICAM1 expression and promotes their susceptibility to cytotoxic T-cells [28]. Interestingly, miR-221 didn't affect ICAM1 expression (Figure 5F), suggesting that ICAM1 is not a target for miR-221. Accordingly, miR-221 had no effect on melanoma cell resistance to T cells (Figure 5H). ICAM1 as a target for miR-221 is a subject of discrepancy; while some have shown that miR-221 targets ICAM1 3′UTR [29, 43], others have failed to do so [48] or didn't find any prediction for miR-221 binding to ICAM1 [28]. In the current work, dual luciferase assays showed that ICAM1 is indeed a target of miR-222 but not of miR-221 (Figure 5I), despite their shared seed region. The differences may stem from the diverse cloning methodology of the 3′UTR (i.e., full or part of the 3′UTR) and of the miR (i.e., mature or precursor) and the different cells used in these experiments. Blocking of ICAM1 in miR-222-OX cell system suggests that at least part of the inhibitory effect conferred by miR-222 is mediated by ICAM1. Since high concentrations of α-ICAM1 Ab led to reduced killing also in miR-222 OX cells, and as microRNAs can modulate the expression of hundreds of different mRNAs [49], it is reasonable to assume that miR-222 targets additional proteins that can potentially affect immune resistance, together with ICAM1. Taken together, these results support an important role for ICAM1 in the immune resistance conferred by ADAR1-regulated miR-222, but not by miR-221.

It has been suggested that at least 6% of all human miRNAs may be subjected to RNA editing [18]. However, we couldn't find any editing events in the pri-miR-222 sequence. Experiments conducted with ADAR1 construct lacking the catalytic domain (ΔCAT-S) show that ADAR1 affects pri-miR-222 and subsequently miR-222 expression independently of its editing properties. Furthermore, the effects of ΔCAT-S on ICAM1 expression and on TIL-mediated killing were similar to the effects exerted by the full ADAR1 protein. These novel findings suggest that ADAR1 regulates melanoma immune resistance independently of RNA-editing, corresponding with very few reports about ADARs editing-independent activities [21, 31, 32, 50]. Nonetheless, we cannot exclude the involvement of RNA editing in immune-tumor interactions, for example by altering the cell's proteome profile, which could directly affect antigenicity, in a similar way somatic mutations affect formation of neoepitopes [51, 52].

Previous studies showed that ADARs can affect miRNAs function at different stages of biogenesis leading to their altered processing and thereby modulate their expression levels in the cell [40, 41, 50] or change the set of miR targets [19]. Our results show that ADAR1 affects miR-222 expression at the pri-miR stage (Figures 7A, 7B). Over-expression of ADAR1 reduced the activity of the miR-222 promoter (Figure 7D), suggesting transcriptional regulation of miR-222 expression. Previous reports have shown that PLZF [47] and proto-oncogene ETS-1 [53] are transcriptional regulators of miR-222 in melanoma by direct binding to its putative regulatory region. Our system failed to show direct binding of ADAR1 to pri-miR-222 suggesting that ADAR1 affects the transcription of miR-222 indirectly. Nonetheless, our negative results could be a result of technical obstacles and thus direct binding of ADAR1 to pri-miR-222 cannot be completely excluded.

The anti-CTLA-4 mAb ipilimumab is an approved treatment for metastatic melanoma. Ipilimumab facilitates an improved generation of effector T cells against the tumor cells [34]. Due to this mechanism of action, it is impossible to predict who will benefit from this treatment. We show that miR-222 retrospectively differentiates between patients that benefited from treatment with ipilimumab from patients that did not. Indeed, miR-222 was expressed at significantly higher levels (2.3 folds) in tumors from patients who did not benefit from ipilimumab. Staining of ICAM1 in tumors from these patients showed a trend towards higher expression of ICAM1 in patients who did benefit from ipilimumab, however these results were not statistically significant, probably due to the small sample size. To confirm these results, prospective studies with larger sample size are warranted. Biomarker analyses were previously reported, indicating on various in-situ “inflammatory” parameters that differentiate between patients benefit or not from ipilimumab [54]. Discerning between two possible mechanisms may not be simplistic: a) lower miR-222 in patients benefiting from ipilimumab is secondary to primary tumor immunogenicity leading to a more inflammatory environment (e.g. IFN) that upregulates ADAR1, causing the reduction observed in miR-222. This could probably further enhance ICAM1 and facilitate the inflammatory environment; b) primary lower miR-222 levels lead to better recognition of melanoma cells by infiltrating lymphocytes due to higher ICAM1 expression, leading to secondary enhanced inflammatory signature. In the current work, we could not find any differences in infiltration and spatial scattering of lymphocytes pre-treatment between patients with clinical benefit from ipilimumab and those without. Similar results were previously reported by Hamid et al. [55]. The recent publication of Snyder et al. [51] indicating in a retrospective analysis that benefit from ipilimumab treatment is linked with higher burden of somatic tumor mutations causing potentially neoepitopes supports the first hypothesis. Notably, in the supplementary data of the comprehensive publication by Ji et al [54], high ICAM1 is reported among the inflammatory signature. Therefore our finding that low miR-222 expression pre-treatment is associated with benefit from ipilimumab fits the currently available data and provides a mechanistic insight with clinical relevance.

These results suggest that miR-222 may serve as a reliable biomarker for the prediction of response to ipilimumab. Given its role in ICAM1 regulation, it might also predict response to other immunotherapies currently investigated such as anti-PD1.

Several cell systems (e.g., ADAR1-KD, ADAR1-p110, miR-222 OX etc.) were used in this study. The differences observed in absolute cytotoxicity between these cell systems may stem from the different vectors. In addition, there is known inter-experimental variability in the absolute TIL cytotoxicity activity due to the nature of these primary cells. It is therefore very hard to provide quantitative extrapolations based on these different experiments of different cell systems. Thus, our conclusions and proposed mechanistic link between ADAR1-miR-222-ICAM1 and immune resistance are based on comparison to the appropriate controls using the same cells and vectors, dose-dependent antibody blocking and effector-to-target ratios.

In conclusion, our group has recently shown that ADAR1 has a fundamental role in regulating the aggressiveness of melanoma, and it is downregulated along melanoma progression [21]. Others have shown that miR-222 expression increases along with melanoma progression, to induce a more tumorigenic phenotype [47]. Induction of ICAM1 in resistant melanoma cells is sufficient to restore the susceptibility of tumor cells to the CTL-mediated death [39]. Together with the new mechanistic findings presented in this report, ADAR1 and miR-222 may serve as good targets for the treatment of melanoma.

## MATERIALS AND METHODS

### Cells and media

The human metastatic melanoma cancer cell lines 624mel (HLA-A*02, -A*03), 526mel (HLA-A*02, -A*03, -B*15, -Cw*03) and 938mel (HLA-A*0101, -A*2402, -B*0702, -B*0801, -Cw*0701, -Cw*0702) were obtained from Dr. Steve Rosenberg (National Cancer Institute, Bethesda, MD), and the C8161 was obtained from Dr. Marry Hendrix (Children's Memorial Research Center, Chicago, IL). Cell lines were maintained in RPMI-1640 medium (Biological Industries, cat#01-100-1A) with supplements and 10% FBS (Biological Industries, cat#04-127-1A). The primary TIL bulk cultures – TIL14 (HLA-A*02, -A*33, -B*1402, -B*2702), TIL51 (HLA-A*02, -A*30, -B*13, -B*39, -Cw*06, -Cw*07) and TIL52 (HLA-A*0201, -B*18, -B*35) (obtained from surgically excised melanoma specimens at our institute) and JKF6 TIL clone (HLA-A*0201, -A*11, -B*15, -B*50, -Cw*03, -Cw*06) obtained from Dr. Steve Rosenberg (National Cancer Institute), were cultured as previously described [11]. 293T cells (ATCC) were maintained in Dulbecco's Modified Eagle's Medium (DMEM) (Biological Industries, cat#01-055-1A) with 10% FBS. The generated stable cell lines were cultured similarly with addition of 1 μg/ml of puromycine (Merck Millipore, cat#540411) or 2mg/ml G418 (Alexis Biochemicals, cat#ALX-380-013) to the culture medium.

### Generation of stable expression cell systems

The ADAR1 knockdown system is based on shRNA oligonucleotides subcloned into pSuper.puro vector. The ADAR1 over-expression and His-tagged constructs were subcloned into pCDNA3.1.neo vector. The miR-222 and miR-221 precursors constructs were subcloned into pQCXIP.puro vector. The detailed list of primers used for the generation of all constructs appears in Supplementary Table S1. Transfections were performed using Turbofect (Fermentas, cat#R0531) according to manufacturer's instructions. Retroviral transductions were performed as previously described [56, 57]. All transfectants were tested routinely for expression.

### RNA isolation and reverse transcription

Total RNA was isolated using Tri Reagent (Sigma-Aldrich, cat#T9424), and cDNA was generated by High-capacity reverse transcriptase kit (Applied Biosystems, cat#4374966) using random hexamer primers or Universal Transcriptor cDNA master (Roche Diagnostics, cat#05893151001), according to manufacturer's instructions. cDNA for miRNAs was generated using TaqMan microRNA custom primers (Applied Biosystems) or Universal cDNA synthesis kit (Exiqon, cat#203301).

### Quantitative real-time PCR (qRT-PCR)

Primers (Sigma-Aldrich) for different genes were designed according to Primer-Express software guidelines (Applied Biosystems). miRNAs expression was tested using custom TaqMan primers (Applied Biosystems) or MicroRNA LNA™ PCR primers (Exiqon). The qRT-PCR reactions were run in triplicates on ABI7500 system utilizing SDS 1.2.3 Software (Applied Biosystems, Carlsbad, CA) or LightCycler480 system (Roche, Basel, Switzerland). Gene transcripts were detected using 2X SYBR Green Master Mix (Applied Biosystems, cat#4309155) or LightCycler480 SYBR Green I Master (Roche, cat#04-887-352). miRNAs transcripts were detected using TaqMan Universal PCR Master Mix (Applied Biosystems, cat#4304437) or SYBR Green master mix (Exiqon, cat#203400, according to manufacturer's instructions. Reactions were normalized to GAPDH, HPRT or U6 endogenous control. Relative expression was calculated using 2^(−ΔΔCt) equation, as previously described [58]. The detailed list of primers used for qRT-PCR appears in Supplementary Table S1.

### LDH cytotoxicity assays

Cytotoxicity assays were performed by measuring lactate dehydrogenase (LDH) release, according to manufacturer's instructions (CytoTox 96, Promega, cat#G1780). Briefly, target cells were co-incubated overnight with effector cells at different E:T ratios in a 96-well plate. 45 min prior to harvesting supernatants, 10 μl of lysis solution was added to a group of wells to obtain maximum LDH release. Plates were centrifuged and 50 μl of supernatants were transferred to a fresh 96-well plate. 50 μl of LDH substrate mix were added to each well and plates were incubated covered at room temperature. After 30 min, 50 μl of stop buffer were added to each well. The LDH release was estimated by using a microplate reader (GloMax, Promega, Madison, WI) at 490 nm. For blocking assays, target cells were pre-incubated for 1 h on ice with different concentrations of anti-Human ICAM1 monoclonal mouse IgG1 antibody (R&D Systems, cat#BBA3) or mouse IgG1 isotype control (BioXCell, cat#BE0083), followed by 9 h cytotoxicity assays. All experiments were performed in triplicate wells. (E:T) represents the ratio between effector (TILs) and target (melanoma cells). Percent of specific lysis of target cells was calculated using the equation:

$$({\text{Experiemental-Effector}}_{\text{Spontaneous}}-{\text{Target}}_{\text{Spontaneous}})/({\text{Target}}_{\text{Maximum}}-{\text{Target}}_{\text{Spontaneous}})\times 100.$$

### CFSE cytotoxicity

Cytotoxicity assays based on CFSE pre-labeling of target cells and PI co-staining after co-incubation with the effector cells were performed using flow cytometry, as previously described [59]. For blocking assays, target cells were pre-incubated for 1 h on ice with mouse anti-human HLA-A2 antibody (Serotec, Oxford, UK) or mouse IgG2b isotype control (Serotec), followed by 5 h cytotoxicity assays. Percent of specific lysis of target cells was determined after subtraction of background. Background signal never exceeded 20%. E:T represents the ratio between effector (TILs) and target (melanoma cells).

### Condition medium and transwell experiments

Conditioned medium (CM) assays were performed by seeding 300K/well ADAR1-KD or Scramble cells in a 6 well plate. After 24 h, CM was collected. TILs were pre-incubated for 1 h with ADAR1-KD or Scramble CM. After 1 h, CFSE-labeled melanoma cells were added to the TILs and co-incubated for 5 h or overnight. Cytotoxicity assay was performed as described above.

Transwell experiments were performed by seeding 50K/well ADAR1-KD or Scramble cells into the upper wells of a modified Boyden chamber (pore size 5 μm) (Costar, cat#3421). 50K CFSE-labeled melanoma cells and given amounts of effector cells were placed in the lower wells below the permeable membrane. After 5 h or overnight incubation, killing rate in the lower well was assessed as described above.

### Quantification of IFN-γ secretion

624mel ADAR1-KD or Scramble cells were co-incubated with TILs for 5 h. Following incubation, the amount of IFN-γ in supernatants was evaluated by standardized enzyme-linked immunosorbent assay (ELISA) (R&D Systems, cat#DY285).

### Evaluation of ZAP70 phosphorylation

TIL14 were mixed at a 5:1 cell ratio with ice cold 624mel ADAR1-KD or Scramble cells. Following gentle vortex stimulations were carried for 10 min using a water-bath at 37°C and terminated with the addition of cold PBS. Cells were immediately pelleted and lysed by triton based lysis buffer supplemented with phosphatase and protease inhibitor cocktails for 30 min on ice. 20 μg of protein from the lysates were used for subsequent Western Blot (WB), as previously described [21]. Membranes were exposed to p-ZAP70Y319 primary antibody (Cell Signaling Technology, cat#C-2701S) overnight at 4°C. For total ZAP70 and load control analysis, membranes were striped by low pH buffer, blocked and exposed to ZAP70 (Santa Cruz Biotechnology, cat#sc-574) and actin antibodies (Santa Cruz Biotechnology, cat#sc-1616) for 60 min at room temperature. Exposure was done by a secondary peroxidase-conjugated anti-rabbit Ab (Santa Cruz Biotechnology, cat#SC-1616R) and standard ECL reagent (Pierce, cat#PIR-34077). Revelation and quantification of WB data was performed using an ImageQuant LAS 500 imager (GE Healthcare) and the image analysis program Image Studio Digits (LI-COR, Lincoln, NE).

### Flow cytometry

Staining for extracellular and intracellular proteins was performed according to standard protocols, as previously described [58, 59]. Gating of cells was performed using FSC vs. SSC. Background fluorescence intensity was set by isotype control or secondary antibody only stained samples. Staining was determined by FACSCalibur instrument (BD Biosciences, San Jose, CA), and data analysis was performed using FlowJo software (Tree Star Inc., Ashland, OR). The following antibodies were used: anti human HLA-ABC Antigen-RPE (W6/32) (DAKO, cat#R7000), Human ICAM1 monoclonal mouse IgG1 antibody (R&D Systems, cat#BBA3), FITC-Kat4C (DAKO, cat#F7112), anti-melanoma (gp100, MART1; Abcam, cat#AB-ab732), Alexa Fluor 488 goat anti-mouse IgG (Life Technologies, cat#A11001), Human CD11a-PE (Beckman Coulter, cat#IM1433U), Mouse IgG1/RPE (DAKO, cat#X0928).

### Western blotting

WB using ADAR1 (Sigma-Aldrich, cat#HPA003890), polyHistidine (R&D Systems, cat#MAB050), and β-actin (MP Biomedicals, cat#0869100) antibodies was performed according to manufacturer's recommendations and as previously described [21].

### Determination of apoptosis

624mel ADAR1-p110 and Mock cells were stained with both annexin V–FITC and PI according to the manufacturer's instructions (eBioscience, cat#BMS500FI). Apoptosis rates and data analysis were determined by flow cytometry as described above.

### Immunoprecipitation-PCR

Procedure was performed as previously described [21]. Briefly, 293T cells were seeded in five 10cm culture dishes and transfected with ADAR1 or Carcinoembryonic antigen-related cell adhesion molecule 1 (CEACAM1) constructs together with miR-222 precursor construct. After 48 h, RNA was extracted from one culture dish using Tri Reagent (Sigma-Aldrich) in order to assess ADAR1, CEACAM1 and pri-miR-222 expression by qRT-PCR as described above. The remaining cells were immunoprecipitated using Dynabeads Protein G (Invitrogen, cat#Dy_10003D) and anti-ADAR (Sigma-Aldrich) or anti-CEACAM1 (MRG1 [57]) antibodies. At the end of the precipitation procedure, RNA was extracted using miRNeasy Kit (Qiagen, cat#217004). Reverse transcription was obtained using Universal Transcriptor cDNA master (Roche). Successful transfection and immunoprecipitation were confirmed by WB. Pri-miR-222 expression following immunoprecipitation was assessed by qRT-PCR.

### Luciferase reporter assay

The 3′UTR of ICAM1 (ICAM1 UTR; ∼1300 bp) was amplified and cloned into psiCheck2 vector (Promega), downstream of the Renilla luciferase gene. The firefly luciferase allowed normalization of Renilla luciferase expression. Three point mutations were inserted into the predicted binding site of miR-221 and miR-222 (ICAM1 UTR MUT) using QuikChange Site-Directed Mutagenesis Kit (Stratagene, cat#200518), according to manufacturer's protocol. Primers used for cloning appear in Table SI. 293T cells were co-transfected with Turbofect (Fermentas) and with (a) 200 ng of miR-221, miR-222, or pQCXIP empty vector (as control); and (b) 20 ng of ICAM1 UTR, ICAM1 UTR MUT or psiCheck2-empty vector. Cells were harvested 48 h after transfection and assayed with Dual Luciferase Reporter Assay System (Promega, cat#1960) according to the manufacturer's instructions.

### Promoter luciferase assay

A DNA fragment containing the putative promoter of miR-222 (∼2000bp upstream of pre-miR-222) was amplified and cloned into pGL4.14 vector (Promega). Primers used for cloning appear in Table SI. 293T cells were transfected with Turbofect (Fermentas) according to manufacturer's instructions and (a) 180ng ADAR1-p110 or Mock construct; (b) 18ng of pGL4.14 empty or pGL4.14 containing miR-222 putative promoter and; (c) 4ng Renilla. After 48 h, cells were lysed and luciferase activity was measured with Dual Luciferase Reporter Assay System (Promega) and normalized to Renilla. The Mock plasmid co-transfected with pGL4.14 empty was considered as control.

### Microarray expression analysis

Melanoma samples were derived from metastatic melanoma patients treated with ipilimumab at Sheba Medical Center (IRB approval in Sheba: 8946-11-smc). Formalin fixed paraffin embedded (FFPE) melanoma tissues were stained with hematoxilin and eosin (H&E) for examination by an expert pathologist. Non-tumor tissue was removed. Total RNA was isolated using miRNeasy FFPE kit (Qiagen, cat# 217504) according to the manufacture guidelines. RNA was used as template to generate a biotin-labeled target that was processed by an Affymetrix GeneChip Instrument System (Affymetrix, Santa Clara, CA) according to manufacturer's recommendations, as previously described [21]. Microarray data are accessible through GEO Series accession number GSE67496 (http://www.ncbi.nlm.nih.gov/geo/query/acc.cgi?acc=GSE674960).

### Determination of lymphocytes infiltration and ICAM1 staining in melanoma specimens

H&E-stained slides of melanoma sections described above, were evaluated by an expert pathologist, blinded to the experimental groups, and categorized for the presence/absence of lymphocytes infiltration and spatial scattering (non-brisk/brisk). Immunohistochemical staining of ICAM1 (Sigma-Aldrich, cat# HPA002126) was performed on 4 μm sections of paraffin-embedded tissues according to standard procedures, as previously described [2121]. Intensity of ICAM1 membrane expression was scored from 0 (negative) to 3 and percentages of expression were defined.

### Statistics

Data were analyzed using the unpaired two-tailed Student's *t* test. In all graphs, error bars represent Standard Error. Asterisks indicate *P* values: *P < 0.05, **P < 0.01, ***P < 0.001.

## SUPPLEMENTARY FIGURES AND TABLE



## References

[1] Berwick M, Erdei E, Hay J (2009). Melanoma epidemiology and public health. Dermatol Clin.

[2] Balch CM, Houghton AN, Sober AJ, Soong S-J (2003). Cutaneous Melanoma.

[3] Sang M, Wang L, Ding C, Zhou X, Wang B, Lian Y, Shan B (2011). Melanoma-associated antigen genes - an update. Cancer Lett.

[4] Hodi FS, O'Day SJ, McDermott DF, Weber RW, Sosman JA, Haanen JB, Gonzalez R, Robert C, Schadendorf D, Hassel JC, Akerley W, van den Eertwegh AJ, Lutzky J, Lorigan P, Vaubel JM, Linette GP (2010). Improved survival with ipilimumab in patients with metastatic melanoma. N Engl J Med.

[5] Robert C, Thomas L, Bondarenko I, O'Day S, M DJ, Garbe C, Lebbe C, Baurain JF, Testori A, Grob JJ, Davidson N, Richards J, Maio M, Hauschild A, Miller WH, Gascon P (2011). Ipilimumab plus dacarbazine for previously untreated metastatic melanoma. N Engl J Med.

[6] Ribas A, Hodi FA, Kefford R, Hamid O, Daud A, Wolchok JD, Hwu W, Gangadhar TC, Patnaik A, Joshua AM, Hersey P, Weber JS, Dronca RS, Zarour HM, Gergich K, Li X (2014). Efficacy and safety of the anti-PD-1 monoclonal antibody MK-3475 in 411 patients (pts) with melanoma. J Clin Oncol.

[7] Hodi FS, Ribas A, Daud A, Hamid O, Robert C, Kefford R, Hwu WJ, Gangadhar TC, Joshua AM, Hersey P, Weber JS, Dronca RS, Perrone AM, Gammage L, Hille D, Xue D (2014). Evaluation of immune-related response criteria (irRC) in patients (pts) with advanced melanoma treated with the anti-PD-1 monoclonal antibody MK-3475. J Clin Oncol.

[8] Hodi FS, Sznol M, Kluger HM, McDermott DF, Carvajal RD, Lawrence DP, Topalian SL, Atkins MB, Powderly JD, Sharfman WH, Puzanov I, Smith DC, Leming PD, Lipson EJ, Taube JM, Anders R (2014). Long-term survival of ipilimumab-naive patients (pts) with advanced melanoma treated with nivolumab (anti-PD-1, BMS-936558, ONO-4538) in a phase I trial. J Clin Oncol.

[9] Brahmer JR, Tykodi SS, Chow LQ, Hwu WJ, Topalian SL, Hwu P, Drake CG, Camacho LH, Kauh J, Odunsi K, Pitot HC, Hamid O, Bhatia S, Martins R, Eaton K, Chen S (2012). Safety and activity of anti-PD-L1 antibody in patients with advanced cancer. N Engl J Med.

[10] Sznol M, Kluger HM, Callahan MK, Postow MA, Gordon RA, Segal NH, Rizvi NA, Lesokhin AM, Atkins MB, Kirkwood JM, Burke MM, Ralabate AL, Rivera AL, Kronenberg SA, Agunwamba B, Feely W (2014). Survival, response duration, and activity by BRAF mutation status of nivolumab (NIVO, anti-PD-1, BMS-936558, ONO-4538) and ipilimumab (IPI) concurrent therapy in advanced melanoma. J Clin Oncol.

[11] Besser MJ, Shapira-Frommer R, Itzhaki O, Treves AJ, Zippel D, Levy D, Kubi A, Shoshani N, Zikich D, Ohayon Y, Ohayon D, Shalmon B, Markel G, Yerushalmi R, Apter S, Ben-Nun A (2013). Adoptive Transfer of Tumor Infiltrating Lymphocytes in Metastatic Melanoma Patients: Intent-to-Treat Analysis and Efficacy after Failure to Prior Immunotherapies. Clin Cancer Res.

[12] Rosenberg SA (2012). Raising the bar: the curative potential of human cancer immunotherapy. Sci Transl Med.

[13] Rosenberg SA, Dudley ME (2009). Adoptive cell therapy for the treatment of patients with metastatic melanoma. Curr Opin Immunol.

[14] Zinshteyn B, Nishikura K (2009). Adenosine-to-inosine RNA editing. Wiley Interdiscip Rev Syst Biol Med.

[15] Maas S, Rich A, Nishikura K (2003). A-to-I RNA editing: recent news and residual mysteries. J Biol Chem.

[16] Levanon EY, Eisenberg E, Yelin R, Nemzer S, Hallegger M, Shemesh R, Fligelman ZY, Shoshan A, Pollock SR, Sztybel D, Olshansky M, Rechavi G, Jantsch MF (2004). Systematic identification of abundant A-to-I editing sites in the human transcriptome. Nat Biotechnol.

[17] Kim DD, Kim TT, Walsh T, Kobayashi Y, Matise TC, Buyske S, Gabriel A (2004). Widespread RNA editing of embedded alu elements in the human transcriptome. Genome Res.

[18] Blow MJ, Grocock RJ, van Dongen S, Enright AJ, Dicks E, Futreal PA, Wooster R, Stratton MR (2006). RNA editing of human microRNAs. Genome Biol.

[19] Kawahara Y, Zinshteyn B, Sethupathy P, Iizasa H, Hatzigeorgiou AG, Nishikura K (2007). Redirection of silencing targets by adenosine-to-inosine editing of miRNAs. Science.

[20] Luciano DJ, Mirsky H, Vendetti NJ, Maas S (2004). RNA editing of a miRNA precursor. RNA.

[21] Nemlich Y, Greenberg E, Ortenberg R, Besser MJ, Barshack I, Jacob-Hirsch J, Jacoby E, Eyal E, Rivkin L, Prieto VG, Chakravarti N, Duncan LM, Kallenberg DM, Galun E, Bennett DC, Amariglio N (2013). MicroRNA-mediated loss of ADAR1 in metastatic melanoma promotes tumor growth. J Clin Invest.

[22] Hartner JC, Walkley CR, Lu J, Orkin SH (2009). ADAR1 is essential for the maintenance of hematopoiesis and suppression of interferon signaling. Nat Immunol.

[23] Cai L, Li Y, Liu F, Zhang W, Huo B, Zheng W, Ding R, Guo J, Zhao Q, Dou K (2010). The influence of ADAR1's regulation on lymphocyte cell function during rejection. Mol Biol Rep.

[24] Markel G, Seidman R, Stern N, Cohen-Sinai T, Izhaki O, Katz G, Besser M, Treves AJ, Blumberg RS, Loewenthal R, Mandelboim O, Orenstein A, Schachter J (2006). Inhibition of human tumor-infiltrating lymphocyte effector functions by the homophilic carcinoembryonic cell adhesion molecule 1 interactions. J Immunol.

[25] Au-Yeung BB, Deindl S, Hsu LY, Palacios EH, Levin SE, Kuriyan J, Weiss A (2009). The structure, regulation, and function of ZAP-70. Immunol Rev.

[26] Jenkins MR, Griffiths GM (2010). The synapse and cytolytic machinery of cytotoxic T cells. Curr Opin Immunol.

[27] Bachmann MF, McKall-Faienza K, Schmits R, Bouchard D, Beach J, Speiser DE, Mak TW, Ohashi PS (1997). Distinct roles for LFA-1 and CD28 during activation of naive T cells: adhesion versus costimulation. Immunity.

[28] Ueda R, Kohanbash G, Sasaki K, Fujita M, Zhu X, Kastenhuber ER, McDonald HA, Potter DM, Hamilton RL, Lotze MT, Khan SA, Sobol RW, Okada H (2009). Dicer-regulated microRNAs 222 and 339 promote resistance of cancer cells to cytotoxic T-lymphocytes by down-regulation of ICAM-1. Proc Natl Acad Sci U S A.

[29] Hu G, Gong AY, Liu J, Zhou R, Deng C, Chen XM (2010). miR-221 suppresses ICAM-1 translation and regulates interferon-gamma-induced ICAM-1 expression in human cholangiocytes. Am J Physiol Gastrointest Liver Physiol.

[30] Galeano F, Tomaselli S, Locatelli F, Gallo A (2012). A-to-I RNA editing: the “ADAR” side of human cancer. Semin Cell Dev Biol.

[31] Nie Y, Ding L, Kao PN, Braun R, Yang JH (2005). ADAR1 interacts with NF90 through double-stranded RNA and regulates NF90-mediated gene expression independently of RNA editing. Mol Cell Biol.

[32] Ota H, Sakurai M, Gupta R, Valente L, Wulff BE, Ariyoshi K, Iizasa H, Davuluri RV, Nishikura K (2013). ADAR1 forms a complex with Dicer to promote microRNA processing and RNA-induced gene silencing. Cell.

[33] Ul Hussain M (2012). Micro-RNAs (miRNAs): genomic organisation, biogenesis and mode of action. Cell Tissue Res.

[34] O'Day SJ, Hamid O, Urba WJ (2007). Targeting cytotoxic T-lymphocyte antigen-4 (CTLA-4): a novel strategy for the treatment of melanoma and other malignancies. Cancer.

[35] McGovern VJ (1975). Spontaneous regression of melanoma. Pathology.

[36] Stewart TJ, Abrams SI (2008). How tumours escape mass destruction. Oncogene.

[37] Yaguchi T, Sumimoto H, Kudo-Saito C, Tsukamoto N, Ueda R, Iwata-Kajihara T, Nishio H, Kawamura N, Kawakami Y (2011). The mechanisms of cancer immunoescape and development of overcoming strategies. Int J Hematol.

[38] Igney FH, Krammer PH (2002). Immune escape of tumors: apoptosis resistance and tumor counterattack. J Leukoc Biol.

[39] Hamai A, Meslin F, Benlalam H, Jalil A, Mehrpour M, Faure F, Lecluse Y, Vielh P, Avril MF, Robert C, Chouaib S (2008). ICAM-1 has a critical role in the regulation of metastatic melanoma tumor susceptibility to CTL lysis by interfering with PI3K/AKT pathway. Cancer Res.

[40] Kawahara Y, Megraw M, Kreider E, Iizasa H, Valente L, Hatzigeorgiou AG, Nishikura K (2008). Frequency and fate of microRNA editing in human brain. Nucleic Acids Res.

[41] Yang W, Chendrimada TP, Wang Q, Higuchi M, Seeburg PH, Shiekhattar R, Nishikura K (2006). Modulation of microRNA processing and expression through RNA editing by ADAR deaminases. Nat Struct Mol Biol.

[42] Valastyan S, Weinberg RA (2011). Roles for microRNAs in the regulation of cell adhesion molecules. J Cell Sci.

[43] Kan AA, van Erp S, Derijck AA, de Wit M, Hessel EV, O'Duibhir E, de Jager W, Van Rijen PC, Gosselaar PH, de Graan PN, Pasterkamp RJ (2012). Genome-wide microRNA profiling of human temporal lobe epilepsy identifies modulators of the immune response. Cell Mol Life Sci.

[44] Lee EJ, Gusev Y, Jiang J, Nuovo GJ, Lerner MR, Frankel WL, Morgan DL, Postier RG, Brackett DJ, Schmittgen TD (2007). Expression profiling identifies microRNA signature in pancreatic cancer. Int J Cancer.

[45] He H, Jazdzewski K, Li W, Liyanarachchi S, Nagy R, Volinia S, Calin GA, Liu CG, Franssila K, Suster S, Kloos RT, Croce CM, de la Chapelle A (2005). The role of microRNA genes in papillary thyroid carcinoma. Proc Natl Acad Sci U S A.

[46] Galardi S, Mercatelli N, Giorda E, Massalini S, Frajese GV, Ciafre SA, Farace MG (2007). miR-221 and miR-222 expression affects the proliferation potential of human prostate carcinoma cell lines by targeting p27Kip1. J Biol Chem.

[47] Felicetti F, Errico MC, Bottero L, Segnalini P, Stoppacciaro A, Biffoni M, Felli N, Mattia G, Petrini M, Colombo MP, Peschle C, Care A (2008). The promyelocytic leukemia zinc finger-microRNA-221/-222 pathway controls melanoma progression through multiple oncogenic mechanisms. Cancer Res.

[48] Suarez Y, Wang C, Manes TD, Pober JS (2010). Cutting edge: TNF-induced microRNAs regulate TNF-induced expression of E-selectin and intercellular adhesion molecule-1 on human endothelial cells: feedback control of inflammation. J Immunol.

[49] Selbach M, Schwanhausser B, Thierfelder N, Fang Z, Khanin R, Rajewsky N (2008). Widespread changes in protein synthesis induced by microRNAs. Nature.

[50] Heale BS, Keegan LP, McGurk L, Michlewski G, Brindle J, Stanton CM, Caceres JF, O'Connell MA (2009). Editing independent effects of ADARs on the miRNA/siRNA pathways. EMBO J.

[51] Snyder A, Makarov V, Merghoub T, Yuan J, Zaretsky JM, Desrichard A, Walsh LA, Postow MA, Wong P, Ho TS, Hollmann TJ, Bruggeman C, Kannan K, Li Y, Elipenahli C, Liu C (2014). Genetic basis for clinical response to CTLA-4 blockade in melanoma. N Engl J Med.

[52] Rizvi NA, Hellmann MD, Snyder A, Kvistborg P, Makarov V, Havel JJ, Lee W, Yuan J, Wong P, Ho TS, Miller ML, Rekhtman N, Moreira AL, Ibrahim F, Bruggeman C, Gasmi B (2015). Mutational landscape determines sensitivity to PD-1 blockade in non-small cell lung cancer. Science.

[53] Mattia G, Errico MC, Felicetti F, Petrini M, Bottero L, Tomasello L, Romania P, Boe A, Segnalini P, Di Virgilio A, Colombo MP, Care A (2011). Constitutive activation of the ETS-1-miR-222 circuitry in metastatic melanoma. Pigment Cell Melanoma Res.

[54] Ji RR, Chasalow SD, Wang L, Hamid O, Schmidt H, Cogswell J, Alaparthy S, Berman D, Jure-Kunkel M, Siemers NO, Jackson JR, Shahabi V (2012). An immune-active tumor microenvironment favors clinical response to ipilimumab. Cancer Immunol Immunother.

[55] Hamid O, Schmidt H, Nissan A, Ridolfi L, Aamdal S, Hansson J, Guida M, Hyams DM, Gomez H, Bastholt L, Chasalow SD, Berman D (2011). A prospective phase II trial exploring the association between tumor microenvironment biomarkers and clinical activity of ipilimumab in advanced melanoma. J Transl Med.

[56] Greenberg E, Hershkovitz L, Itzhaki O, Hajdu S, Nemlich Y, Ortenberg R, Gefen N, Edry L, Modai S, Keisari Y, Besser MJ, Schachter J, Shomron N, Markel G (2011). Regulation of cancer aggressive features in melanoma cells by microRNAs. PLoS One.

[57] Ortenberg R, Sapir Y, Raz L, Hershkovitz L, Ben Arav A, Sapoznik S, Barshack I, Avivi C, Berkun Y, Besser MJ, Ben-Moshe T, Schachter J, Markel G (2012). Novel immunotherapy for malignant melanoma with a monoclonal antibody that blocks CEACAM1 homophilic interactions. Mol Cancer Ther.

[58] Markel G, Ortenberg R, Seidman R, Sapoznik S, Koren-Morag N, Besser MJ, Bar J, Shapira R, Kubi A, Nardini G, Tessone A, Treves AJ, Winkler E, Orenstein A, Schachter J (2010). Systemic dysregulation of CEACAM1 in melanoma patients. Cancer Immunol Immunother.

[59] Markel G, Seidman R, Cohen Y, Besser MJ, Sinai TC, Treves AJ, Orenstein A, Berger R, Schachter J (2009). Dynamic expression of protective CEACAM1 on melanoma cells during specific immune attack. Immunology.

