# Supplementary material for: A novel immune resistance mechanism of melanoma cells controlled by the ADAR1 enzyme
Source: Oncotarget. 2015 Aug 21;6(30):28999–9015. doi: 10.18632/oncotarget.4905 (PMC4745707; doi:10.18632/oncotarget.4905)
Supplement: Supplementary file 1 [file oncotarget-06-28999-s001.pdf]

SUPPLEMENTARY FIGURES AND TABLE

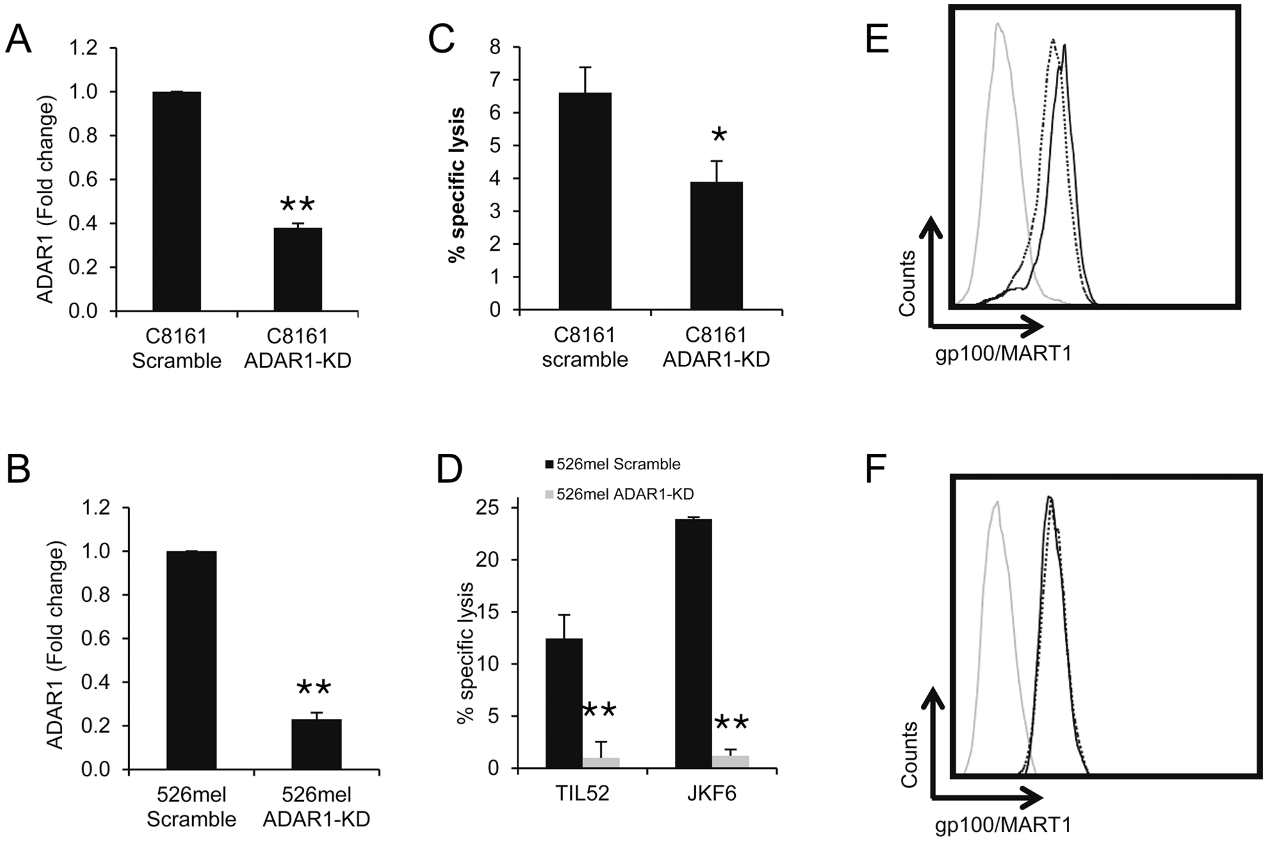

Supplementary Figure S1: ADAR1 regulates melanoma immune resistance. ADAR1 mRNA levels were assessed by qRT-PCR and normalized to GAPDH expression in C8161 **A.** and 526mel **B.** melanoma cells. CFSE-labeled C8161 ADAR1-KD and Scramble cells were co-incubated with TIL14 **C.** and CFSE-labeled 526mel ADAR1-KD and Scramble cells were co-incubated with TIL2 or JKF6 **D.** for 5 hours. Specific lysis of melanoma cells was assessed by flow cytometry. ADAR1-KD (dotted line) and Scramble (black line) cells were stained with anti-melanoma (gp100/MART1) antibody in C8161 **E.** and 526mel **F.** melanoma cells. Intracellular expression was analyzed by flow cytometry.

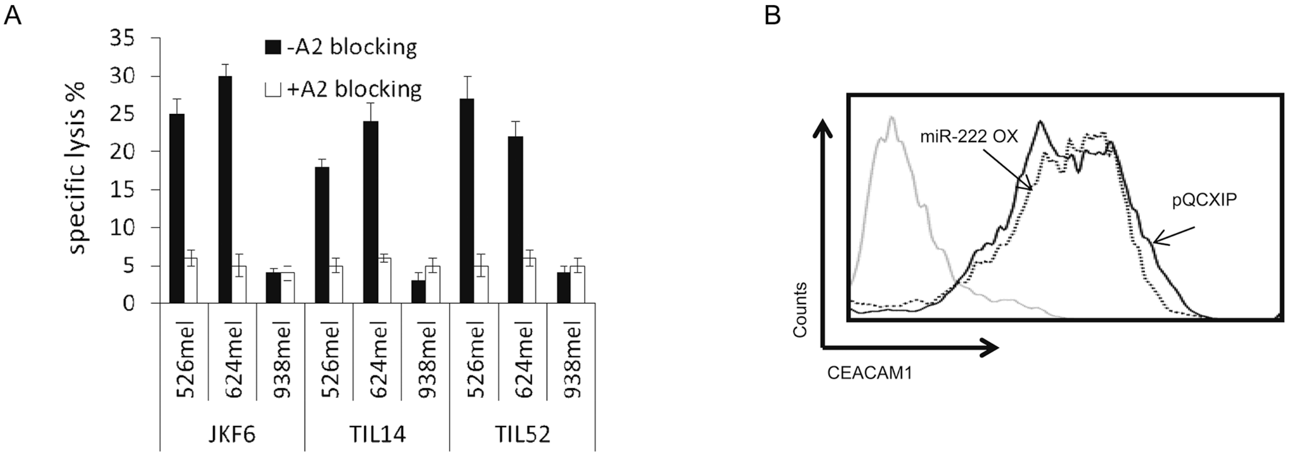

Supplementary Figure S2: A. TIL-mediated cytotoxicity is HLA-A2 restricted. CFSE-labeled 526mel, 624mel and 938mel cells were incubated with isotype control (-A2) or HLA-A2 antibody. After 1h, cells were co-incubated with JKF6 for 5h (E:T - 10:1). Specific lysis of melanoma cells was assessed by flow cytometry. **B.** miR-222 over-expression does not affect CEACAM1 protein expression. CEACAM1 protein expression in miR-222 OX (dotted line) and pQCXIP (black line) cells was analyzed by extracellular flow cytometry staining using FITC-Kat4C antibody (Dako, Glostrup, Denmark).

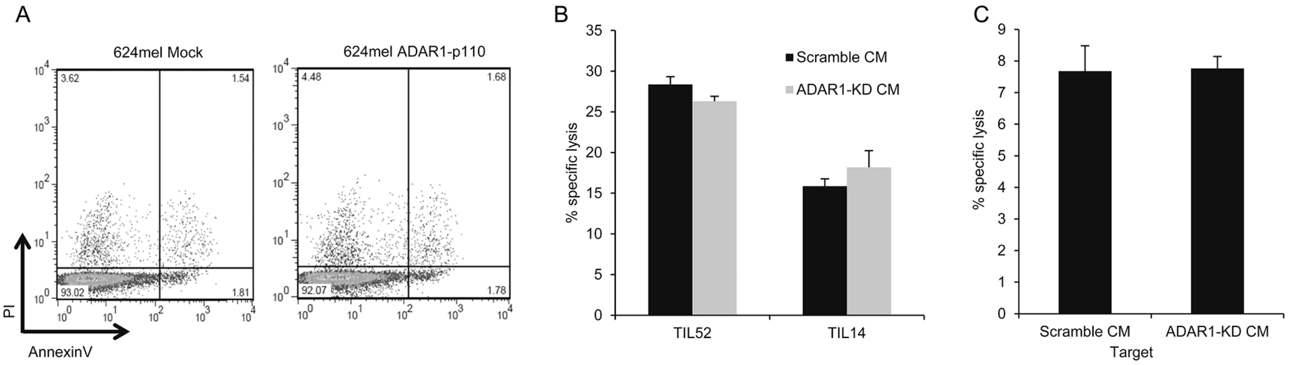

Supplementary Figure S3: ADAR1 does not induce spontaneous apoptosis. Spontaneous apoptosis of ADAR1-p110 and Mock cells was determined by AnnexinV and PI co-staining and analyzed by flow cytometry. **A. ADAR1-mediated regulation of melanoma immune resistance is dependent on cell-cell interaction.** TILs were pre-incubated with conditioned media (CM) from 624mel ADAR1-KD or Scramble cells. After 1h, the cells were co-incubated with non-manipulated melanoma cells; TIL52 or TIL14 were co-incubated with 526mel cells over-night **B.** and TIL52 were co-incubated with C8161 cells for 5 h **C.** Specific lysis of melanoma cells was assessed by flow cytometry.

Supplementary Table S1: List of primers used

Gene	Forward primer	Reverse primer
Generation of constructs primers		
ADAR1 p110	5′- GCTAAGCTTGCCGCCACCA TGGCCGAGATCAAGGAGAAA	5′-GCTCTCGAGCTATACTGGGC AGAGTTAAAAGTTCTTTTCCTCC
ΔCAT-S	5′- GCTAAGCTTGCCGCCACCA TGGCCGAGATCAAGGAGAAA	5′-GATCTCGAGCTAATGGT GATGGTGATGATGCACTGG GGTTACCTCTGTGAA
miR-222-precursor	5′-AAGAATGCGGCCGCATGAT CCAAAGAAAATGTGCA	5′-CGGAATTCGACTGCCCA ATAATCTCTCTCA
miR-221-precursor	5′-GTACGCGGCCGCCCCAGCA TTTCTGACTGTTG	5′-GCGAATTCCCTCCTGGA AAACAGTTATTCAG
miR-222 promoter	5′-CCGCTCGAGACAAGGGGCA AAAGACCA	5′-CCCAAGCTTTGGGTGAT CCTTTGCCTT
ICAM1 UTR	5′-CCGCTCGAGACCTATCCCG GGACAGG	5′- ATGCGCGGCCGCACTAA CACAAAGGAAGTCTGGG
ICAM1 UTR MUT	5′-GGGCCTTTGTGTTTTGAGG ATAGACATGTCTATGGAGGGCCACTT	5′-AAGTGGCCCTCCATAGA CATGTCTATCCTCAAAACACAAAGGCCC
qRT-PCR primers		
ADAR1	5′-ACAGCCAAAGACACTCCCTCTC	5′-GGCTCAGCATGGCTATCTGG
ADAR1-HIS	5′ GCTCTCCGTGTCTTGATTGGG	5′ TGGTGATGGTGATGATGCACTG
ICAM1	5′-TGCAGACAGTGACCATCTACAGC	5′-TCACCTCGGTCCCTTCTGAG
GAPDH	5′-TGCACCACCAACTGCTTAGC	5′-GGCATGGACTGTGGTCATGAG
HPRT	5′-TGACACTGGCAAAACAATGCA	5′-GGTCCTTTTCACCAGCAAGCT
Pri-miR-222	5′-AGCTGCTGGAAGGTGTAGGT	5′-TCTCAGGACACTGAAGCAGAAG
